# Omental Infarction: The Great Impersonator

**DOI:** 10.7759/cureus.1940

**Published:** 2017-12-13

**Authors:** Kevin G Buell, Alexandra Burke-Smith, Vishal Patel, Josef Watfah

**Affiliations:** 1 School of Medicine, Imperial College London; 2 General Surgery, Northwick Park Hospital

**Keywords:** acute cholecystitis, omental infarction, acute abdomen

## Abstract

A 58-year-old female presented to the emergency department with intermittent right upper quadrant pain and nausea. On examination, the patient was tender and Murphy’s sign was elicited. A presumptive diagnosis of acute cholecystitis was made but an ultrasound of the abdomen revealed a thin-walled gallbladder without calculi. A computed tomography (CT) scan of the abdomen and pelvis demonstrated fat stranding involving the greater omentum and the right paracolic gutter. The patient was diagnosed with a focal omental infarction and underwent emergency laparoscopic surgery. Intraoperatively, the thickened and infarcted omental segment was dissected off the abdominal wall, liver, and mesocolon and removed through the umbilical port site using an Endo Catch™ (Covidien Ltd, Dublin, Republic of Ireland). This paper presents a rare case of omental infarction and illustrates how it can mimic the classic presentation of acute cholecystitis. The literature around the incidence, pathogenesis, and management of omental infarction is reviewed and presented to the reader.

## Introduction

Acute abdominal pain accounts for 7% of all emergency department visits and yet the evaluation of this common presentation remains complex [1]. There are many causes of acute abdominal pain, giving rise to the possibility of misdiagnosis. Omental infarction was first reported in 1896 and today is acknowledged to be an uncommon cause of the acute abdomen. This case report illustrates how omental infarction can mimic common causes of right-sided abdominal pain, principally acute cholecystitis, and reviews the literature around the presentation, incidence, pathogenesis, and management of this rare condition.

## Case presentation

A 58-year-old female presented to the emergency department with a two-day history of abdominal pain. The pain was severe, intermittent in nature, and was confined to the right upper quadrant (RUQ). There were no exacerbating or alleviating factors and the pain was not related to the consumption of food. The patient felt nauseated and had vomited once. She was not on any regular medication and had no past surgical or medical history. She did not consume alcohol and did not smoke.

On examination she was afebrile with a heart rate of 84 beats per minute and blood pressure of 105/72 mmHg. Her abdomen was soft, but she was tender in the right upper quadrant of the abdomen with a positive Murphy’s sign. Her full blood count, clotting, renal and bone profile serology were within normal limits with a C-reactive protein (CRP) of 23 mg/L.

**Figure 1:**
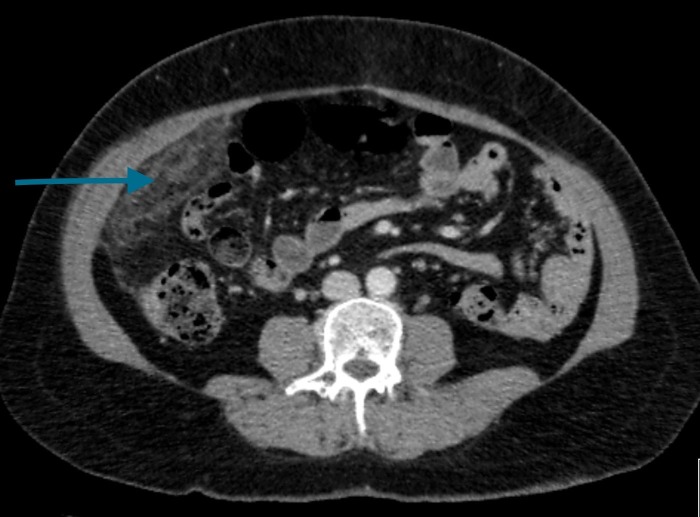
Computed tomography scan - axial view demonstrating fat stranding around the greater omentum.

The initial assumption was that the patient had acute cholecystitis and she was admitted for further imaging. A chest X-ray was unremarkable and had no free air under the diaphragm. An ultrasound of the abdomen revealed a thin walled gallbladder without any calculi and no intrahepatic or extrahepatic duct dilation. Two days from her admission, the patient remained tender in the RUQ and CRP had increased to 70 mg/L. A computed tomography (CT) scan of the abdomen and pelvis was performed to further investigate the cause of the pain. It demonstrated fat stranding and a small amount of fluid around the greater omentum and within the right paracolic gutter (Figure 1).

**Figure 2:**
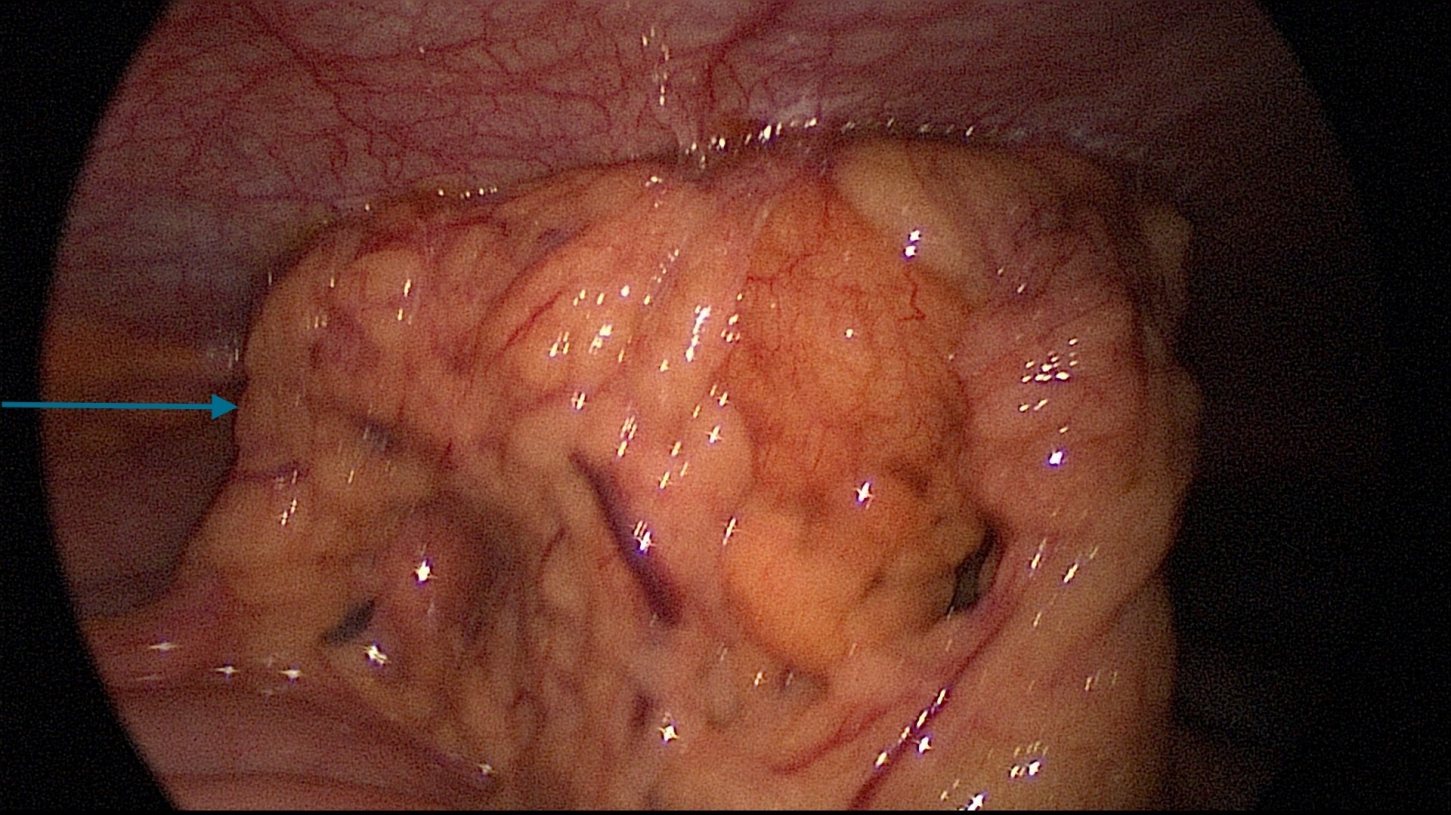
Intraoperative picture demonstrating an infarcted omentum adherent to the abdominal wall.

The patient was diagnosed with a focal omental infarction. She underwent an emergency laparoscopy. Intraoperatively, the greater omental segment was adherent to the abdominal wall and the lateral aspect of the liver, appeared thickened, and was partially infarcted (Figure 2).

The abnormal segment of the greater omentum was dissected off the abdominal wall, liver, and mesocolon and was removed with an Endo Catch™ (Covidien Ltd, Dublin, Republic of Ireland) through the umbilical port. The appendix and gallbladder were both identified to be normal.

The patient had a successful recovery postoperatively without complications and was discharged home two days after the procedure. Her pain had significantly improved.

## Discussion

The greater omentum is a double layer folding of the peritoneum, composed of connective tissue, fat, and lymphatics that originates from the greater curvature of the stomach and duodenum. It extends inferiorly to cover the lower abdominal organs by wrapping itself around the majority of the intestines. It has previously been described as “the abdominal policeman”, referring to its ability to migrate and limit the spread of intra-abdominal infection. Although this theory has subsequently been disproven, the greater omentum’s ability for adhesion formation, neovascularisation and infection defence combined with its abundant availability has stemmed great interest with respect to its application in defect reconstructive surgery in the abdomen and pelvis. It is perhaps fitting that primary omental pathology is rare, yet metastatic invasion, omental cysts, omental torsion, and omental infarction have all been reported in the literature.

Omental infarction can be classified into two categories: primary and secondary as described in the landmark paper by Leitner, et al. [2]. In both types, infarction can occur in the absence or presence of torsion of the greater omentum. The infarction results from venous stasis, thrombosis, and haemorrhagic necrosis that are subsequently demonstrated through histological evidence of venous congestion, thrombosis, haemorrhage, and fat cell necrosis [3]. Primary omental infarction occurs spontaneously without any immediate aetiology and has consequently been named idiopathic segmental infarction of the greater omentum (ISIGO). Anatomical variations such as malformation, local variations in fat distribution, and redundant omental veins may predispose patients to ISIGO [4]. It is hypothesised that the torsion and subsequent infarction can be triggered by compression of the greater omentum between the liver and abdominal wall following local trauma, excessive exercise, occupational vibration, and increased intra-abdominal pressure secondary to excessive straining or coughing. Secondary omental infarct has an identifiable cause such as neoplasms and inflammatory conditions causing adhesions between the omentum and pathological foci [5]. Inguinal hernias can also entrap the omentum at the inguinal ring causing omental strangulation. Infarcted omentum is subsequently found as a content of the inguinal hernia during surgery.

The incidence of omental infarction has yet to be accurately determined, but over 400 cases have been documented in the literature to date. The condition is most prevalent in children, followed by the 40-50-year-old age category whereby the male to female ratio is 2:1. The principal feature in the clinical presentation is abdominal pain. Previous case reports have characterised the pain as sudden in its onset, non-radiating, and most frequently located on the right hand side.The predisposition for right-sided side abdominal pain has been reported as high as 88% of all presentations and is thought to be caused by the omentum’s longer length and greater mobility on the right hand side in comparison to the left [5]. Associated features such as altered bowel habit and vomiting are infrequent.

Omental infarction can mimic the classic presentation of an acute abdomen. On clinical examination, patients demonstrate signs of local peritonism. Haematological and biochemical serology can show a non-specific inflammatory response but these investigations may equally fall within normal limits [6]. In cases of omental infarction caused by inguinal herniation, the patient can present with acute scrotal pain instead of the previously discussed acute abdomen. As illustrated in our case, the presentation of omental infarction was mistaken for a more common cause of right sided abdominal pain, i.e. acute cholecystitis. Other differentials to be considered should include appendicitis, viscus perforation, and colitis amongst others. In women, gynaecology differentials must be considered such as an ovarian cyst rupture and ectopic pregnancy.

In the past, omental infarction was diagnosed intraoperatively, but with new advances in imaging technology, it is becoming more readily detectable outside the surgical theatres. CT is the imaging modality of choice and the appearance of all types of omental pathology can broadly be classified into four categories: omental caking (the infiltration of omental fat by soft tissue), fat stranding with smudged appearance, cystic masses, and discrete nodules [7]. Omental infarction is most frequently represented by fat stranding adjacent to the bowel wall and in particular, fat stranding that is disproportionate to the degree of bowel wall thickening. Differentials for this radiological finding include appendicitis and diverticulitis [8]. In addition to CT, ultrasound may equally aid clinical decision-making and be used for the exclusion of biliary stones as a likely differential. Although it remains the second choice of imaging modality, ultrasound can equally diagnose omental infarction through the appearance of non-compressible and increased echogenic mass in the omental fat [9].

There are two approaches to managing omental infarction: conservative and laparoscopic excision. Open laparotomy is becoming outdated due its more invasive nature in comparison to the conservative or laparoscopic approach. The choice of management remains controversial and should be decided on an individual basis as guided by the clinical and imaging findings. Conservative management is composed of oral analgesia, anti-inflammatory medication, and prophylactic antibiotics. Early surgical intervention has the advantage of reduced incidence of omental necrosis, abscess formation, and development of adhesions. With prompt discharge following a laparoscopic approach, surgery can also reduce the overall length of stay. In comparison, symptoms can persist with conservative management for weeks, with the mean time to symptoms resolution being 13.5 days [10]. However, the surgical approach is equally associated with perioperative risks in anaesthesia, intraoperative complications, and postoperative morbidity caused by the surgery. Conservative management remains a viable option for non-operative candidates due to comorbidities. In our case report, a laparoscopic approach was favoured due to the severity of the pain.

## Conclusions

After exclusion of common causes of right-sided abdominal pain, omental infarction should be considered amongst the differential diagnoses. CT can accurately identify a focal area of fat stranding around the omentum, the most frequent radiological finding. Although management of this rare condition should always employ a case-by-case approach, the authors report successful early laparoscopic excision of a primary omental infarction on a previously healthy 58-year-old female.
